# Soluble Expression and Efficient Purification of Recombinant Class I Hydrophobin DewA

**DOI:** 10.3390/ijms22157843

**Published:** 2021-07-22

**Authors:** Sang-Oh Ahn, Ho-Dong Lim, Sung-Hwan You, Dae-Eun Cheong, Geun-Joong Kim

**Affiliations:** 1 Department of Biological Sciences, College of Natural Sciences, Chonnam National University, Yongbong-ro, Buk-gu, Gwangju 61186, Korea; repaul2001@gmail.com (S.-O.A.); decheong01@gmail.com (D.-E.C.); 2 Center for Industrialization of Agricultural and Livestock Microorganisms, 241 Cheomdangwahak-ro, Jeongeup-si 56212, Jeollabuk-do, Korea; eastlake@cialm.or.kr; 3 Biomedical Research Center, Chonnam National University, Convergence Science Building (M2), Suite 301-1 264, Seoyang-ro, Hwasun-eup, Hwasun-gun 58128, Jeollanam-do, Korea; shyou@cncure.co.kr

**Keywords:** ramp tag (RT), soluble expression, recombinant hydrophobin DewA, aqueous two-phase separation (ATPS), isopropyl alcohol (IPA)

## Abstract

Hydrophobins are small proteins (<20 kDa) with an amphipathic tertiary structure that are secreted by various filamentous fungi. Their amphipathic properties provide surfactant-like activity, leading to the formation of robust amphipathic layers at hydrophilic–hydrophobic interfaces, which make them useful for a wide variety of industrial fields spanning protein immobilization to surface functionalization. However, the industrial use of recombinant hydrophobins has been hampered due to low yield from inclusion bodies owing to the complicated process, including an auxiliary refolding step. Herein, we report the soluble expression of a recombinant class I hydrophobin DewA originating from *Aspergillus nidulans,* and its efficient purification from recombinant *Escherichia coli*. Soluble expression of the recombinant hydrophobin DewA was achieved by a tagging strategy using a systematically designed expression tag (ramp tag) that was fused to the N-terminus of DewA lacking the innate signal sequence. Highly expressed recombinant hydrophobin DewA in a soluble form was efficiently purified by a modified aqueous two-phase separation technique using isopropyl alcohol. Our approach for expression and purification of the recombinant hydrophobin DewA in *E. coli* shed light on the industrial production of hydrophobins from prokaryotic hosts.

## 1. Introduction

Hydrophobins are small proteins with an amphipathic structure that were initially isolated from *Schizophyllum commune* [1]. This family of proteins is mainly composed of 100–150 amino acids, which are frequently found in the mycelia of fungi. Hydrophobins provide a hydrophobic surface layer through self-assembly on the hydrophilic hypha of growing mycelium, when monomers are secreted on the surface of growing mycelia. As a result, hydrophobins serve to coat the surface of the hyphae due to the amphiphilic nature of the protein [2,3,4]. In addition, hydrophobins not only enable hyphae to effectively respond to environmental changes but also function as a shield between the cell wall and air layer or at the interface between the cell wall and a solid surface during sporulation, fruiting body development, and infection structure formation through host invasion [5].

Hydrophobins typically have four disulfide bonds originating from eight cysteine residues [6], which play an important role in stabilizing the amphipathic three-dimensional structure that imparts activity to the hydrophobin similar to that of a surfactant. Hydrophobins are typically divided into two classes (class I and II) depending on hydropathy, solubility, and the structure formed during self-assembly. Both class I and II can form an amphipathic monolayer at a hydrophilic–hydrophobic interface [7,8]. However, class I hydrophobins form amyloid-like rodlets that are insoluble at a hydrophilic–hydrophobic interface, whereas class II hydrophobins form a monolayer with high solubility at a hydrophilic–hydrophobic interface [9,10,11,12,13]. Due to the above features, hydrophobins have been spotlighted in the biomaterial industry [14]. Accordingly, utilizing hydrophobins in cosmetics or food and beverages requiring a stable and uniform foam has been well studied [15]. In addition, hydrophobins have received increased attention in various industries as an innovative next-generation material, including use as a medical coating agent, and as a functional particle in nanostructures applied to the living body [15,16,17].

Currently, various production methods, including a recombinant technique using bacteria, yeast, and plant cells as expression hosts, or a fermentation method using the original producer fungus have been attempted [18,19,20,21,22]. Although several reports described successfully soluble expression in *Pichia pastoris*, recombinant hydrophobins produced in bacteria, especially *E. coli*, as inclusion bodies need to be preprocessed for further utilization. In this case, a denaturation/refolding process is required to obtain a functional protein, which results in increased cost and time for the mass production of hydrophobins. For example, it was reported by BASF SE in 2009 that two recombinant class I hydrophobins, H*Protein A (yaaD-DewA-His_6_), and H*Protein B (truncated yaaD-DewA-His_6_), can be produced in adequate amounts for industrial application, using bacteria as a host. Unfortunately, in this case, hydrophobins were also produced in an inclusion body form. Until now, there have been no case reports in which recombinant hydrophobins have been successfully overexpressed in a soluble form [23]. Additionally, a mechanical process using HPLC or a two-phase separation method (e.g., ATPS; aqueous two-phase separation) using a surfactant have mainly been used for purifying hydrophobins. However, this takes a long time and requires additional cost due to complex purification procedures and use of chemicals that are not easy to handle [24,25]. Therefore, these issues require urgent attention.

Here, we report that soluble expression of a recombinant hydrophobin DewA was successfully achieved by using a combination strategy of N-terminal fusion with a rationally designed expression tag (ramp tag) and deletion of the DewA signal peptide. In addition, we verified that the hydrophobin expressed was soluble and efficiently purified by ATPS using IPA. Thus, time-consuming and low-efficiency protein refolding and purification steps can be avoided.

## 2. Results

### 2.1. Preparation and Screening of Ramp Tags for Soluble Expression of Hydrophobin DewA

In our previous study, a method to prepare target gene-specific (tailor-made) ramp tags was developed to facilitate the overexpression of several recombinant proteins [26]. Unlike the general method of codon optimization or deoptimization for difficult-to-express proteins, it is possible to increase the expression level or solubility of the target protein by merely fusing a ramp tag to the N-terminus of the target protein, without changing the original codons of the open reading frame (ORF). The ramp tag comprises a set of rare codons, deduced from codon usage preference in line with copy numbers of tRNA genes in the genome of *E*. *coli* K12 and those present in the target gene sequence. These codons are then intentionally located at the 5′-end of the target ORF, thereby allowing the tRNAs of these rare codons to be primarily recruited and then reused by ribosomes for translation of the same codon in subsequent positions, as described previously [27]. The resulting ramp tag typically consists of DNA sequences (tandem arrangement of rare codons) encoding 1–20 amino acids.

**Figure 1:**
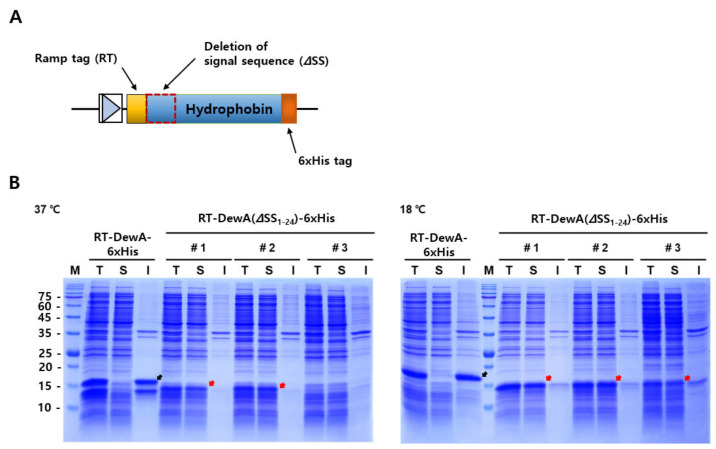
Schematic diagram of the DNA construct designed for the soluble expression of hydrophobin DewA and protein expression behavior of a recombinant hydrophobin DewA with or without the signal sequence. (**A**) Design scheme of the DNA construct used for the soluble expression of recombinant hydrophobin DewA. The rationally designed ramp tag was N-terminally fused, and 6× His tags were fused at the C-terminal region for Western blot analysis. The deleted signal sequence (ΔSS) of DewA is represented as a dotted box. (**B**) SDS-PAGE analyses (15 μg of proteins) of soluble recombinant hydrophobin expression at two different culture temperatures. Protein expressions of three independent clones (#1–3) were induced with 0.2 mM isopropyl β-D-1-thiogalactopyranoside (IPTG) at 37 °C (left panel) or at 18 °C (right panel). M, protein marker; T, total fraction; S, soluble fraction; I, insoluble fraction. Black arrows indicate insoluble expression of hydrophobin RT5-DewA-6xHis, whereas red arrows indicate soluble expression of hydrophobin RT5-DewA(ΔSS_1-24_)-6xHis, wherein DewA(ΔSS_1-24_) represents that the entire predicted signal sequence (total 24 residues) was deleted. The molecular mass of RT5-DewA-6His and RT5-DewA(ΔSS_1-24_)-6×His are 15.7 and 13.4 kDa, respectively.

Considering the above-mentioned criteria, five specific ramp tags were initially prepared by making a rare codon list according to the codon usage preference of the host cell, *E*. *coli* BL21 (DE3). The DNA sequence of the target gene *dewA* was converted into codons, and the frequency and position at which rare codons of the above list appeared in the *dewA* ORF were analyzed. Finally, we collected and arranged these rare codons, randomly or in order of appearance in the target ORF (Figure 1A). The resulting ramp tag DNA sequences, and their corresponding amino acid sequences, were fused to the N-terminal region of hydrophobin DewA and are listed in Table 1.

To induce overexpression and/or soluble expression of the difficult-to-express protein hydrophobin DewA, without codon optimization or directed evolution, five ramp tags were designed and fused with the *DewA* ORF at the 5′-terminal region using PCR, as reported previously [26]. The resulting recombinant plasmids (pET24a_RT-DewA-6xHis) were transformed into *E. coli* BL21 (DE3), and then protein expression was induced with 0.2 mM IPTG. As expected, we observed an increase of approximately 3-fold in the expression levels for two of the ramp tags, RT3 and RT5 (data not shown). There were marginal changes in the expression levels of the other three tags. Looking at the solubility of recombinant proteins with ramp tags, almost all proteins (>95%) were detected in insoluble fraction when the protein expression was induced at 37 °C (see recombinant protein with RT5, RT5-DewA-6xHis in the left panel of Figure 1B). Recombinant protein expression with ramp tag RT3 showed a similar result. These results suggest that N-terminal fusion of the ramp tag did not have any beneficial effects on the soluble expression of recombinant hydrophobin DewA, despite the considerable increase in expression.

We further designed 22 ramp tags, RT6 through RT27 (sequences not shown), based on the above criteria. Unexpectedly, all further recombinant proteins with 22 ramp tags showed no promising results for solubility. Based on this observation, we decided to additionally delete the DNA sequence of the putative signal peptide of hydrophobin DewA as this sequence was not functional in *E. coli* and also did not delete in cytoplasm. Thus, inappropriate folding and/or proteolytic degradation likely occurred, thereby leading to negative effects on the solubility and/or expression of the target protein, hydrophobin DewA, via terminally consecutive fusion with the ramp tag. To test the possibility of alleviating the negative effects of the signal sequence on the solubility of hydrophobin DewA, the recombinant plasmid, pET24a_RT5-DewA-6xHis with the ramp tag RT5 (CTTCACAGTCCTAATCCC), was arbitrarily selected based on the relatively upregulated protein expression when compared to the remaining clones. We then attempted to remove the signal sequence by PCR, using a set of primers (Table 2).

### 2.2. Construction and Expression Analyses of a Ramp Tag-Fused Hydrophobin DewA Lacking the Signal Sequence

Considering previous reports regarding the signal sequence of class I hydrophobins [28,29], and our signal sequence prediction, we constructed a recombinant expression vector, pET24a_RT5-DewA (ΔSS_1-24_)-6xHis, by PCR. The deleted signal sequence of hydrophobin DewA is shown in Table 2.

To analyze whether deletion of the signal sequence can induce hydrophobin DewA expression in the soluble fraction, the recombinant protein lacking the signal sequence (RT5-DewA (ΔSS_1-24_)-6xHis) was induced with IPTG under two different culture temperatures, 18 and 37 °C, at 250 rpm for 3 h. The recombinant clone carrying pET24a_RT5-DewA-6xHis was cultured as a control under the same conditions. SDS-PAGE analysis demonstrated that when expression was induced at 37 °C, the expressed amount (>98%) of recombinant hydrophobin DewA in the soluble fraction was significantly higher than that in the insoluble fraction. Thus, most of the recombinant hydrophobin was expressed in a soluble form when the ramp tag was fused with signal sequence-deleted hydrophobin DewA (Figure 1B, left panel). In addition, we showed that when the recombinant protein, RT5-DewA (ΔSS_1-24_)-6xHis, was induced at 18 °C, the expression was further increased as compared to protein induction at 37 °C. Further, most of the expressed protein (>96%) was detected in the soluble fraction (Figure 1B, right panel). In comparison, the control protein, DewA (ΔSS_1-24_)-6xHis without the RT5 ramp tag, was expressed in the insoluble fraction. Thus, we concluded that the recombinant class I hydrophobin, DewA, was expressed mainly in a soluble form due to a combination of N-terminal ramp tag fusion and the deletion of the signal sequence, regardless of differences in the culture temperature. An additional approach for the expression of recombinant hydrophobin DewA with a different length of deleted signal sequence, RT5-DewA (ΔSS_1-18_)-6xHis, showed a somewhat improved solubility when compared with that of the recombinant protein RT5-DewA-6xHis (data not shown). After repeated confirmation by retransforming the plasmid into freshly made competent cells, DNA sequencing also confirmed the correct fusion of the ramp tag with the *DewA* ORF lacking the signal sequence. Based on these observations, we finally selected pET24a_RT5-DewA (ΔSS_1-24_)-6xHis for further analyses.

### 2.3. Purification of DewA by Aqueous Two-Phase Separation Using Isopropyl Alcohol

**Figure 2:**
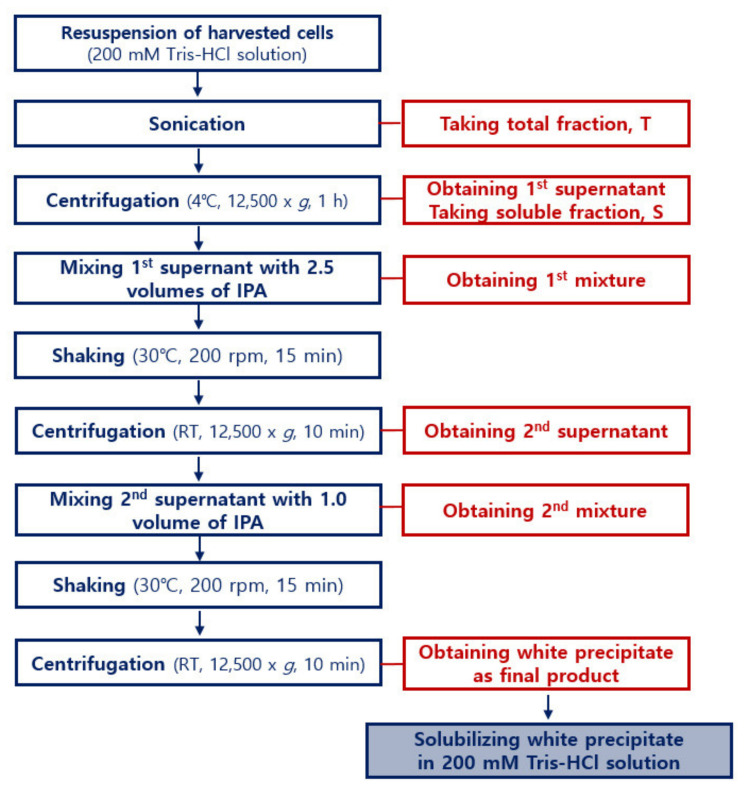
Detailed flowchart of the ATPS purification process for soluble recombinant hydrophobin DewA. This process was established by experimental modifications of known processes in the prior publications. The flowchart shows that the purification procedure uses only one reagent, that is, IPA.

ATPS is a liquid-liquid fractionation technique that has gained interest, both in industry and academia, because of its great potential in the separation, purification, and enrichment of proteins, membranes, viruses, enzymes, nucleic acids, and other biomolecules [24,25,30]. Thus, we chose ATPS as a simple method for purification of the soluble form of recombinant DewA. To this end, we combined and partly modified well-known protocols to optimize the ATPS/IPA procedure for easy and efficient purification of recombinant hydrophobin DewA [24,30,31]. The resulting procedure is illustrated in Figure 2.

**Figure 3:**
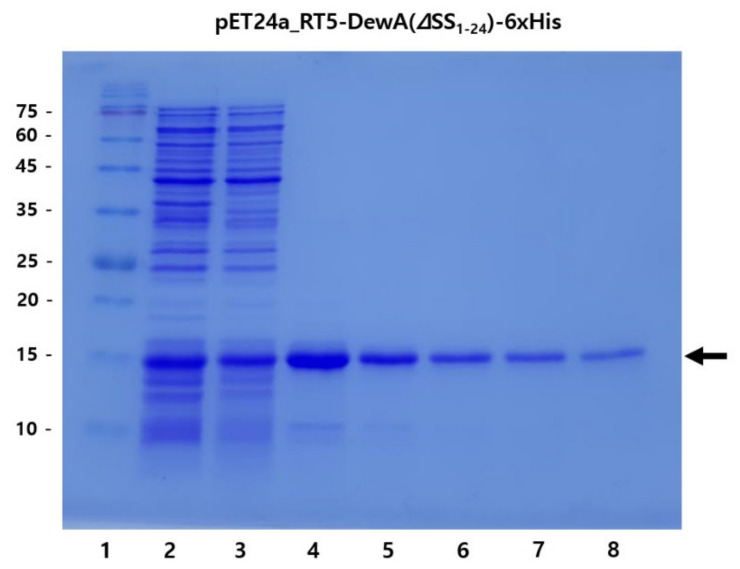
SDS-PAGE analysis of recombinant hydrophobin DewA purified by ATPS using IPA. Lane 1, protein size marker; 2, total fraction; 3, soluble fraction; 4, final white precipitate solubilized in 1 mL of 200 mM Tris-HCl buffer (pH 8.0) buffer following purification; 5–8, samples obtained by sequential dilution of previous sample (lane 4) with 1 mL of the same buffer. The arrow indicates the purified RT5-DewA(ΔSS_1-24_) protein.

During the purification procedure, we prepared seven samples (total fraction, soluble fraction, the solubilized final white precipitate in 1 mL of 200 mM Tris-HCl (pH 8.0) buffer following purification, and serial diluted purified protein samples using the same buffer) to confirm the yield and purity of recombinant hydrophobin DewA using ATPS/IPA. The results are shown in Figure 3.

As shown in Figure 3, SDS-PAGE analysis showed a clear band for the purified recombinant hydrophobin DewA, with over 90% purity. Obligately, 80% or more of the protein in the soluble fraction (>250–400 mg/L) was also observed in the purified fraction (lane 4), thereby proving ATPS an efficient purification method for soluble recombinant hydrophobin DewA. This result strongly implies that the combination of N-terminal ramp tag fusion and deletion of the signal sequence facilitate soluble expression of the recombinant class I hydrophobin DewA, and easy purification thereof can be achieved by ATPS using IPA.

To investigate the applicability of several organic solvents other than IPA, recombinant hydrophobin DewA was also purified as described above. Consequently, there was no comparable or considerable yield, or efficiency even when the conditions (varied protein concentration, treatment time, and buffer) established here were modified (data not shown). We also attempted to purify recombinant hydrophobin DewA using the well-known Triton X-114 as a nonionic surfactant for ATPS, as reported in related work [32,33]. SDS-PAGE of the resulting proteins also showed no considerable results, even under various testing conditions.

## 3. Discussion

There is no doubt about the importance of a soluble expression system, and efficient purification of versatile proteins, such as hydrophobins. Hydrophobins are amphipathic proteins and thus have the ability to spontaneously self-assemble into robust rodlets at hydrophilic–hydrophobic interfaces. Therefore, they have steadily received attention as promising biomaterials that can be used for various purposes through surface modification. To date, many studies have been conducted to realize soluble expression and easy purification of recombinant hydrophobins. However, there are no reports regarding soluble expression of recombinant hydrophobins. For efficient purification, there have been attempts to purify hydrophobin by ATPS using C1–C3 alcohol [31] from concentrated fermentation broth.

This study provides a useful approach for expressing hydrophobins in a soluble form. The combination of N-terminal ramp tag fusion, and deletion of the signal sequence renders possible the expression of recombinant soluble hydrophobin DewA (Figure 1). Although the ramp tag was originally developed to overexpress recombinant fusion proteins [26], promising soluble expression of difficult-to-express proteins has been frequently observed. This, however, is not the case for the expression of soluble protein with only a ramp tag. When the ramp tag fusion approach was used solely, expression of soluble recombinant hydrophobin DewA was not achieved, both at 37 and 18 °C following induction with IPTG (RT5-DewA-6xHis; Figure 1B). However, when the signal sequence was deleted, we showed that solubility of recombinant hydrophobin DewA could be achieved both at 37 and 18 °C (RT5-DewA (ΔSS_1-24_)-6xHis; Figure 1B).

Unfortunately, the molecular mechanism driving the soluble expression of the recombinant hydrophobin DewA using this combination strategy remains poorly understood. We can only presuppose that the combination strategy may have a certain influence on the solubility of the recombinant hydrophobin DewA by altering the N-end rule, thus affecting minimum free energy (MFE), tRNA adaptation index (tAI), translation initiation rate (TIR), and so on. However, our results on the soluble expression of recombinant hydrophobin DewA can be reasonably generalized to at least class I hydrophobins, as most hydrophobins belonging to the same class have common features, such as the arrangement of cysteine residues, hydropathy, position of hydrophobic residues, solubility, and the self-assembly structural properties [34]. Considering these points, further studies are required to elucidate the mechanisms underlying the soluble expression of difficult-to-express class I hydrophobin proteins.

This study also provides an efficient method for purifying the highly expressed recombinant hydrophobin DewA protein in a soluble form. The method established here, ATPS using IPA, provides an efficient route for purifying recombinant hydrophobins, as shown in Figure 2 and Figure 3. This method is highly reproducible, resulting in a yield of over 80% and approximately 90% purity. The coprecipitated impurities were confirmed to be proteolytic fragments of recombinant hydrophobin DewA, which might be avoided by treatment with a protease inhibitor cocktail during sonication. This method was not readily modified for use with alternative commonly used solvents, such as methanol, ethanol, and isobutyl alcohol, because recombinant hydrophobin DewA was not comparably purified when ATPS was carried out under varying conditions when using organic solvents other than IPA. Additionally, using Triton X-114, a nonionic surfactant that has previously been used in ATPS, also failed to purify recombinant hydrophobin DewA.

Although the soluble expression and easy purification of recombinant hydrophobin DewA paves an alternative pathway for the production of difficult-to-express hydrophobins, functional studies, such as evaluation of the coating ability (contact angle measurement) and stability, emulsifying capacity, and ability to modify surfaces, using various tools, including microscopy (fluorescence and/or electron microscopy), remain to be validated [35,36,37]. As a preliminary attempt to determine the coating ability of recombinant hydrophobin DewA, Ponceau staining and contact angle measurements were conducted following treatment on a solid PDMS surface, under varying concentrations and salt conditions. As shown in Figure S1, the structural integrity of the purified RT5-DewA (ΔSS_1-24_) by ATPS was checked by using SEC (size exclusion chromatograph), and the resulting profile showed a possibility that this protein interacted with each other to form multimers in a buffer containing 200 mM Tris-HCl (pH 8.0). Additionally, the purified protein was stable for up to 1 month. We also tentatively attempted to access the functionality of RT5-DewA (ΔSS_1-24_) as a surface modifier through drop shape analyses (Figure S2A,B). Both figures illustrated the behaviors of droplets on hydrophobin non-coated/coated hydrophilic surface (CG, cover glass), hydrophobic surface (CCD, cell culture dish), and superhydrophobic surface (PDMS, polydimethylsiloxane). In this experiment, BSA was also used as a control. Although the purified protein remained stable for up to one month in a low-temperature (4 °C) storage stability test and some positive interpretations through the attempts are possible, however, these attempts did not provide convincing evidence that recombinant hydrophobin DewA containing a ramp tag was functional. We are currently redesigning our strategy to remove the ramp tag after purification, with a specific protease. This work could guarantee soluble expressed recombinant hydrophobin DewA, as a promising candidate for the broad application of versatile hydrophobins.

In conclusion, we showed that a combination strategy of N-terminal fusion with rationally designed ramp tags and deletion of the predicted signal sequence of class I hydrophobin DewA allows for its soluble expression and efficient purification by ATPS using IPA. Although further characterization of the purified protein without the ramp tag remains to be performed, we anticipate that this study will be a stepping stone for breaking the hurdle, “Inclusion body formation followed by refolding for the production of recombinant hydrophobin.”

## 4. Materials and Methods

### 4.1. Strains, Plasmids, and Culture Conditions

The *E*. *coli* strain used for gene cloning and maintenance in this study was XL1-Blue (*recA1 endA1 gyrA46 thi-1 hsdR17 supE44 relA1* lac F’[*proAB laclqZM15* Tn*10*(Tetr)c]) (Stratagene, La Jolla, CA, USA). *E*. *coli* BL21 (DE3) (ompT gal dcm lon hsdSB(rB-mB-) λ (DE3 [lacI, lacUV5-T7p07, ind1, sam7, nin5]) (New England Biolabs, Hitchin, UK) was used as a host for the expression of recombinant hydrophobins. The pET24a backbone vector was used for sub-cloning and expression of the recombinant hydrophobins (Novagen Inc., Madison, WI, USA).

Recombinant *E. coli* was cultured aerobically in Luria-Bertani (LB) medium (10 g/L of tryptone, 5 g/L of yeast extract, and 10 g/L of NaCl) supplemented with 50 µg/mL kanamycin on a rotary shaker (220 rpm) at 18 or 37 °C. Cell growth was routinely monitored by measuring optical density (OD) at 600 nm using a spectrophotometer (UV-1700, Shimadzu, Japan). Recombinant proteins were induced by adding 0.2 mM IPTG at the early exponential phase of cell growth (OD_600_ of approximately 0.4–0.6).

### 4.2. Gene Cloning and Manipulation

The gene sequence encoding class I hydrophobin, DewA, was obtained from GenBank (Gene ID: 2869124, NCBI GI number: 67902037) and commercially synthesized (Bioneer Inc., Dea-Jeon, Korea). To amplify the corresponding DNA fragment, a set of primers listed in Table 1, and Phusion^®^ High-Fidelity DNA Polymerase (New England Biolabs, Hitchin, UK) were used for polymerase chain reaction (PCR). The resulting DNA was purified using a DNA Clean-Up System (Promega, Madison, WI, USA). Restriction enzyme recognition sites added to each primer were used for cloning into the plasmid digested with the same restriction enzymes. Alternatively, cloning was also performed using an In-fusion cloning kit (New England Biolabs, Hitchin, UK) when required.

To induce overexpression, and/or soluble expression of the recombinant hydrophobin DewA, several ramp tags were designed according to the principle described in a previous report [26], and are summarized in Table 1. A brief procedure was described in the results section of this work. The ramp tag was fused to the N-terminus of the gene encoding DewA by PCR. The deleted N-terminal signal sequence of DewA was either determined by a reported result [28,29], or bioinformatically deduced by in silico prediction using SignalP software (http://www.cbs.dtu.dk/services/SignalP, accessed on 4 October 2016). The reported and predicted signal sequences deleted here are summarized in Table 2. The specific deletions of the signal sequences were carried out using conventional PCR and then the signal sequences were fused with ramp tag 5 (RT5) by PCR using a set of synthetic primers.

### 4.3. Expression and Analyses of the Soluble Fraction

To analyze the expression and solubility of the ramp tag fused recombinant hydrophobin DewA, with or without the signal sequence, each plasmid was transformed into *E*. *coli* BL21 (DE3), and then inoculated onto a solid LB medium containing 50 µg/mL of kanamycin and cultured at 37 °C for 12 h. Thereafter, a single colony was inoculated into 3.5 mL of the same liquid medium and pre-cultured at 37 °C at 220 rpm for 5–6 h, following which 100 µL of the pre-culture were re-seeded in 3.5 mL of fresh LB medium. This was then cultured at 37 °C and 220 rpm, and when the absorbance (OD_600_) reached 0.4–0.6, 0.2 mM IPTG was added to induce protein expression. The culture was then incubated for a further 3 h at 250 rpm. For comparative analyses, protein expression was monitored at 18 °C under the same conditions.

The expressed recombinant hydrophobin DewA was recovered from the bacterial culture as follows: (a) 3.5 mL of each culture medium was centrifuged at 4 °C and 10,000× *g* for 10 min, and the resulting pellet was suspended in 200 mM Tris-HCl (pH 8.0) buffer and then lysed by ultrasonication using a pulse of 2 s for 20 s; (b) the cell lysate was centrifuged at 4 °C and 12,500× *g* for 30 min, thereby obtaining a supernatant from which cell debris was removed. Both samples (a) and (b) were used for SDS-PAGE to analyze protein expression in the total and soluble fraction, respectively. The residual fraction following the centrifugation of cell lysate indicated an insoluble protein fraction. All protein samples were analyzed by SDS-PAGE on a 15% (*w*/*v*) polyacrylamide gel. Gels were then stained with Coomassie Brilliant Blue dye and the protein concentration determined using a Bradford assay, with bovine serum albumin as protein standard.

### 4.4. Purification of Recombinant Protein by ATPS Using IPA

Recombinant hydrophobin DewA expressed in the soluble form was purified by ATPS using IPA as follows: (a) a culture medium in which recombinant hydrophobin DewA was expressed was centrifuged at 4 °C and 12,500× *g* to harvest cells; (b) the harvested cells were resuspended in 200 mM Tris-HCl (pH 8.0) buffer and sonicated to lyse the cells, and the total fraction sample was harvested; (c) the lysed cells were then centrifuged at 4 °C and 12,500× *g* for 1 h to obtain the first supernatant, and a soluble fraction sample was taken; (d) a volume of isopropyl alcohol greater than 2.5 times that of the first supernatant was slowly added to the first supernatant to obtain mixture 1; (e) mixture 1 was shaken at 200–250 rpm at 30 °C for 15 min; (f) following this, mixture 1 was centrifuged at room temperature at 12,500× *g* for 10 min to obtain a second supernatant; (g) isopropyl alcohol, the volume of which was the same as that of the second supernatant, was slowly added to the second supernatant to obtain mixture 2; (h) mixture 2 was shaken at 30 °C at 200–250 rpm for 15 min; and (i) following shaking, the mixture 2 was centrifuged at room temperature at 12,500× *g* for 10 min to separate recombinant hydrophobin DewA. The resulting white precipitate was solubilized in 1 mL of 200 mM Tris-HCl (pH 8.0) buffer. When required, 1 mL of the same buffer was further added (to a final volume of 4 mL) to fully solubilize the precipitated protein. The resulting samples were analyzed by SDS-PAGE for purity and yield.

## Figures and Tables

**Table 1:** Designed ramp tags and their corresponding amino acid sequences.

Ramp Tag (RT)		
Name	DNA Sequence	Amino Acid Sequence
RT1	AGTCCTAATCACCCGGGA	SPNHPG
RT2	AGTCCTCCGCACCTTCCC	SPPHLP
RT3	CTTCCCAGTCCTAATCAC	LPSPNH
RT4	AGTCCTAATCCCCCGTCC	SPNPPS
RT5	CTTCACAGTCCTAATCCC	LHSPNP

**Table 2:** Nucleotide sequences of primers and *DewA* with or without the signal peptide.

Name	Nucleotide Sequence
DewA NdeI forward primer	ATCATATGCTTCACAGTCCTAATCCCAAGAACGCGAAGCTGGCC
DewA XhoI reverse primer	ATACTCGAGTTAGTGGTGGTGGTGGTGGTGCTCAGCCTTGGTACCGGCG
RT5-DewA	CTTCACAGTCCTAATCCCCGCTTCATCGTCTCTCTCCTCGCCTTCACTGCCGCGGCCACCGCAACCGCCCTCCCGGCCTCTGCCGCAAAGAACGCGAAGCTGGCCACCTCGGCGGCCTTCGCCAAGCAGGCTGAAGGCACCACCTGCAATGTCGGCTCGATCGCTTGCTGCAACTCCCCCGCTGAGACCAACAACGACAGTCTGTTGAGCGGTCTGCTCGGTGCTGGCCTTCTCAACGGGCTCTCGGGCAACACTGGCAGCGCCTGCGCCAAGGCGAGCTTGATTGACCAGCTGGGTCTGCTCGCTCTCGTCGACCACACTGAGGAAGGCCCCGTCTGCAAGAACATCGTCGCTTGCTGCCCTGAGGGAACCACCAACTGTGTTGCCGTCGACAACGCTGGCGCCGGTACCAAGGCTGAG
RT5-DewA (ΔSS_1-24_)	CTTCACAGTCCTAATCCCAAGAACGCGAAGCTGGCCACCTCGGCGGCCTTCGCCAAGCAGGCTGAAGGCACCACCTGCAATGTCGGCTCGATCGCTTGCTGCAACTCCCCCGCTGAGACCAACAACGACAGTCTGTTGAGCGGTCTGCTCGGTGCTGGCCTTCTCAACGGGCTCTCGGGCAACACTGGCAGCGCCTGCGCCAAGGCGAGCTTGATTGACCAGCTGGGTCTGCTCGCTCTCGTCGACCACACTGAGGAAGGCCCCGTCTGCAAGAACATCGTCGCTTGCTGCCCTGAGGGAACCACCAACTGTGTTGCCGTCGACAACGCTGGCGCCGGTACCAAGGCTGAG

Start codon is intentionally excluded, the RT5 sequence is shaded in yellow, and the signal sequence is shaded in green. Restriction enzyme sites are highlighted in grey.

## Data Availability

No new data were created or analyzed in this study. Data sharing is not applicable to this article.

## Supplementary Materials

The following are available online at https://www.mdpi.com/article/10.3390/ijms22157843/s1.
