# Supplementary material for: Soluble Expression and Efficient Purification of Recombinant Class I Hydrophobin DewA
Source: Int J Mol Sci. 2021 Jul 22;22(15):7843. doi: 10.3390/ijms22157843 (PMC8345945; doi:10.3390/ijms22157843)
Supplement: Supplementary file 1 [file ijms-22-07843-s001.zip › ijms-1261752-supplementary.pdf]

Supplementary Materials:

Soluble Expression and Efficient Purification of Recombinant Class I Hydrophobin DewA

Sang-Oh Ahn [1,†], Ho-Dong Lim [2,†], Sung-Hwan You [3], Dae-Eun Cheong [1] and Geun-Joong Kim [1,*]

1 Department of Biological Sciences and Research Center of Ecomimetics, College of Natural Sciences, Chonnam National University, Yongbong-ro, Buk-gu, Gwangj 61186, Korea; repaul2001@gmail.com (S.-O.A.); decheong01@gmail.com (D.-E.C.)
2 Center for Industrialization of Agricultural and Livestock Microorganisms, 241 Cheomdangwahak-ro, Jeongeup-si 56212, Jeollabuk-do, Korea; eastlake@cialm.or.kr
3 Biomedical Research Center, Chonnam National University, Convergence Science Building (M2), Suite 301-1 264 Seoyang-ro, Hwasun-eup, Hwasun-gun, Jeonnam 58128, Korea; shyou@cncure.co.kr
* Correspondence: gjkim@chonnam.ac.kr; Tel.: +82-62-530-3403
† These authors contributed equally to this work.

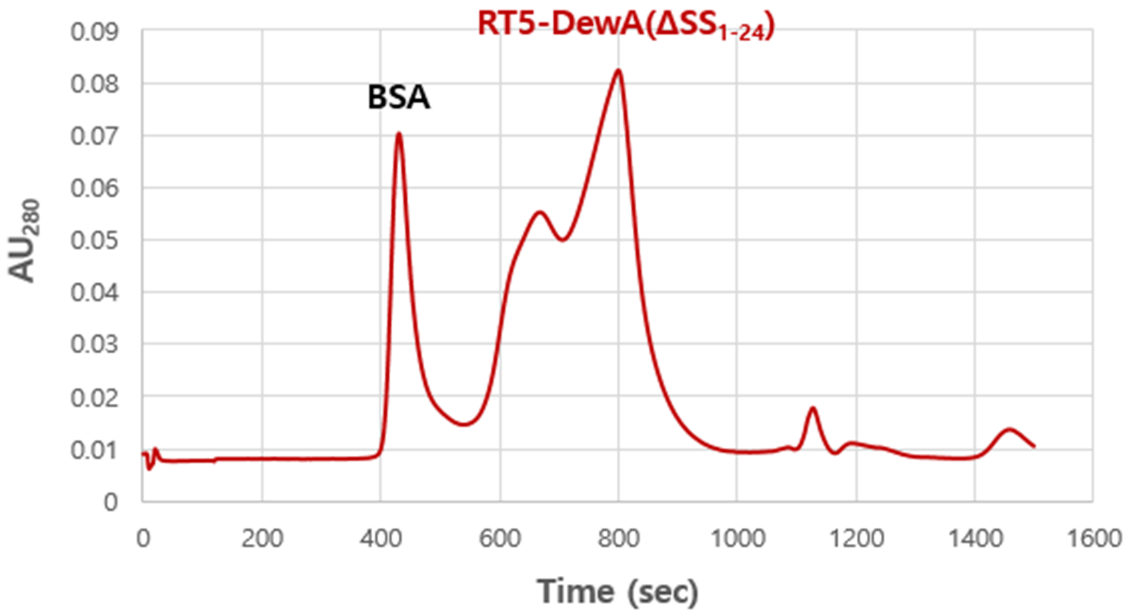

Figure S1. The elution profile of the recombinant protein RT5-DewA (ΔSS$_{1-24}$) by FPLC (BioLogic DuoFlow, Bio-Rad) using a Superose 12 10/300 (GE Healthcare) column. BSA (66 kDa) was used as an internal standard. Two or three major overlapped bands corresponding to the molecular mass ranging from monomer (13 kDa) to multimers (37 kDa) were detected.

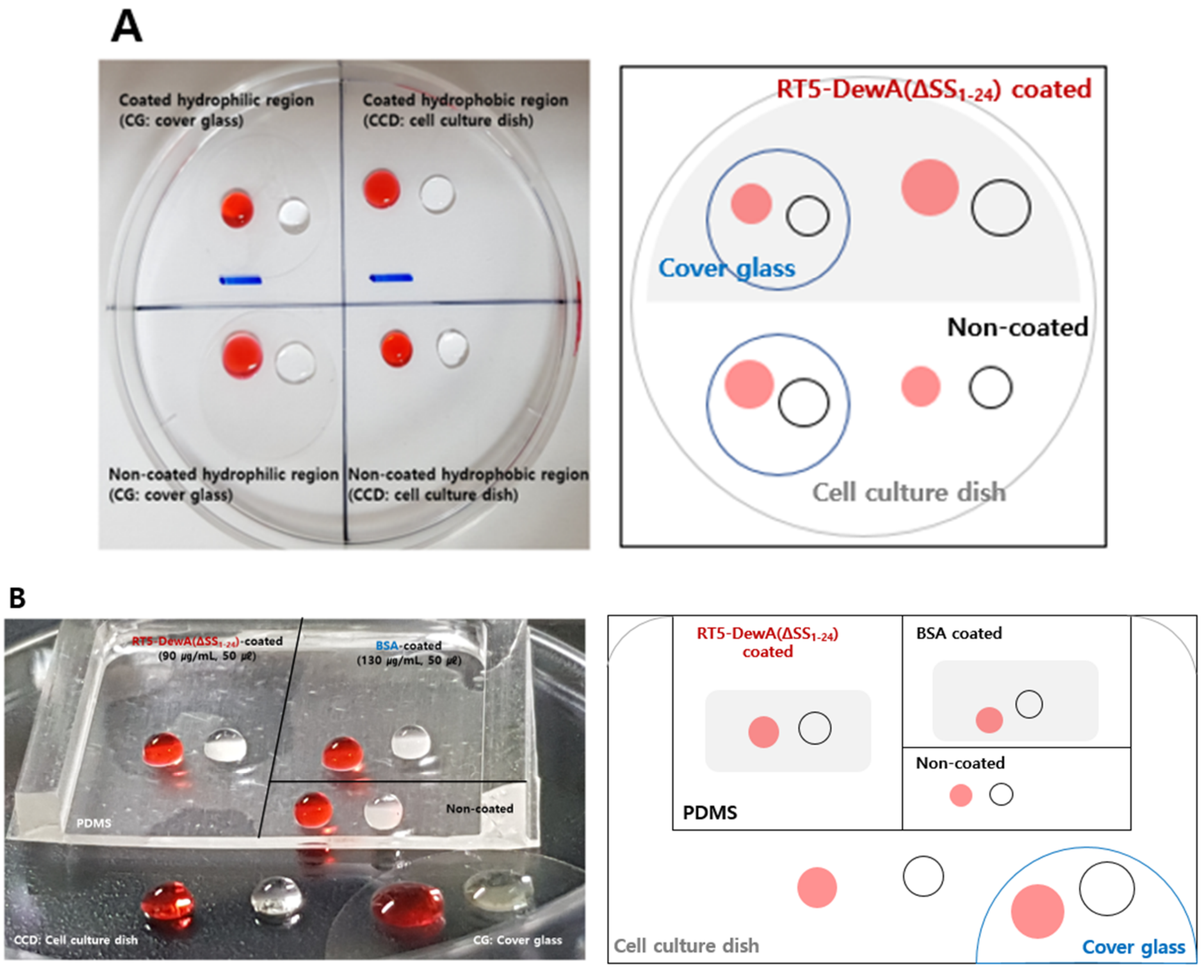

Figure S2. Attempts to access the functionality of the purified protein RT5-DewA(ΔSS_{1-24}) through drop shape analysis. For the analyses of drop shape, the purified RT5-DewA(ΔSS_{1-24}) protein (50 µl) was simply coated by spreading or spraying on the solid surface and then dried, according to the reported procedure in the related research (see ref. 37 of main text and Winandy, L. *et al*., Scientific Reports 2019, 9, 6264). After coating, 10 µl of the water droplet was dropped on the coated and non-coated surfaces, and the resulting shapes were analyzed in terms of sphericity and diameter. BSA was also used as a control. (A) Shapes of water droplets on RT5-DewA(ΔSS_{1-24})-coated cover glass (hydrophilic surface) and cell culture dish (hydrophobic surface). In a schematic representation (right panel), blue circle indicates cover glasses placed in cell culture dish (gray circle). Small black and red circles indicate water droplet (DDW) only and colored water droplet with red dye (ponceau S) to facilitate observation, respectively. Grey shaded region represents RT5-DewA(ΔSS_{1-24}) coated region. Scale bar is 5 mm. (B) Shapes of water droplets on hyper-hydrophobic surface (PDMS). All used circles in this schematic representation have the same meaning as in Figure S2A. RT5-DewA(ΔSS_{1-24})- and BSA-coated regions are represented by gray shades in each rectangle. Cell culture dish and cover glass were used as control surfaces. In this figure, the sphericity of water droplets on hydrophobin coated hydrophilic (or hydrophobic) surface is distinctly (or partially) different from that of non-coated surface due to surface modification by RT5-DewA(ΔSS_{1-24}) coating.
